# Reactive Oxygen Species Mediate Isoalantolactone-Induced Apoptosis in Human Prostate Cancer Cells

**DOI:** 10.3390/molecules18089382

**Published:** 2013-08-05

**Authors:** Azhar Rasul, Jun Di, Faya Martin Millimouno, Mahadev Malhi, Ichiro Tsuji, Muhammad Ali, Jiang Li, Xiaomeng Li

**Affiliations:** 1 The Key Laboratory of Molecular Epigenetics of MOE, Institute of Genetics and Cytology, Northeast Normal University, Changchun 130024, China; 2 Dental Hospital, Jilin University, Changchun 130041, China; 3 Jilin Province People’s Hospital, Changchun 130021, China; 4 Department of Public Health, Tohoku University, Sendai 980-8576, Japan; 5 Institute of Molecular Biology and Biotechnology, Bahauddin Zakariya University, Multan 60800, Pakistan

**Keywords:** prostate cancer, isoalantolactone, apoptosis, reactive oxygen species

## Abstract

Isoalantolactone, a medicinal plant-derived natural compound, is known to induce apoptosis in various cancer cell lines. However, its effect on apoptosis in prostate cancer cells has not been addressed. Thus, we examined the effects of isoalantolactone on prostate cancer cells. It was found that isoalantolactone inhibits growth of both androgen-sensitive (LNCaP) as well as androgen-independent (PC3 and DU-145) prostate cancer cells in a dose-dependent manner. Furthermore, our results indicate that isoalantolactone-induced apoptosis in prostate cancer PC3 cells is associated with the generation of ROS and dissipation of mitochondrial membrane potential (Δψm). In addition, isoalantolactone triggers apoptosis in prostate cancer cells via up-regulation of Bax, down-regulation of Bcl-2, survivin, and significant activation of caspase-3. Isoalantolactone-induced apoptosis is markedly abrogated when the cells were pretreated with *N*-acetylcysteine (NAC), a specific ROS inhibitor, suggesting that the apoptosis-inducing effect of isoalantolactone in prostate cancer cells is mediated by reactive oxygen species. These findings indicate that isoalantolactone induces reactive oxygen species-dependent apoptosis in prostate cancer cells via a novel mechanism involving inhibition of survivin and provide the rationale for further *in vivo* and preclinical investigation of isoalantolactone against human prostate cancer.

## 1. Introduction

Prostate cancer is one of the most prevalent malignancie*s* and the second leading cause of cancer-related deaths among men in the United States, with similar trends in many Western countries [1]. It accounts for 28% of all male cancers and 10% of male cancer-related deaths [2]. Prostate cancer incidence has increased strikingly in many Asian countries in the past two decades [3]. Prevailing treatment options have limited therapeutic success in human prostate cancer, therefore, there is considerable emphasis on identifying novel natural products that selectively induce apoptosis and growth arrest in prostate cancer cells without cytotoxic effects in normal cells [4].

Over the last decade, many reports have revealed that phytochemicals targeting ROS metabolism can selectively kill cancer cells by raising the level of ROS above a toxic threshold. Since cancer cells show higher levels of endogenous ROS compared with normal cells, the toxic threshold can be easily achieved in cancer cells [5,6]. In the current study, we carried out high throughput screening of a compound library from Chinese herbs, using the prostate cancer cell lines, androgen-sensitive (LNCaP) as well as androgen-independent (PC3 and DU-145), in the presence or absence of NAC, a specific ROS inhibitor. This screening strategy helped us to identify natural anticancer compounds targeting ROS mediated apoptosis in prostate cancer cells. Isoalantolactone, a natural compound that belongs to the sesquiterpene lactone family, was identified as a potent growth inhibitor of prostate cancer cells during screening. Sesquiterpene lactones, due to their anti-neoplastic and anti-inflammatory activity, have attracted considerable attention in pharmacological research [7,8]. Isoalantolactone has been reported to have a wide spectrum of biological effects, including anti-fungal and anthelmintic activities [9,10].

Furthermore, isoalantolactone has known anti-proliferative effects against variety of cancer cells, such as colon, melanoma, ovary, lung, and leukemia [11,12]. A previous study also documented that isoalantolactone induced apoptosis in leukemia HL-60 cells [12]. However, the cytotoxic effects of isoalantolactone on human prostate cancer cells and its mechanism of action remained unexplored. Therefore, in the present study, we investigated whether isoalantolactone could inhibit growth of both androgen-sensitive (LNCaP) as well as androgen-independent (PC3 and DU-145) human prostate cancer cell lines with different p53 status. In addition, we have also sought to determine the molecular mechanism(s) underlying isoalantolactone’s effect by investigating the modulation of apoptosis-related proteins and reactive oxygen species (ROS) accumulation. Results demonstrated that isoalantolactone effectively inhibited the proliferation of prostate cancer cells through induction of apoptosis, which is mediated through ROS generation, mitochondrial dysfunction, down-regulation of survivin, and activation of caspase-3.

## 2. Results and Discussion

### 2.1. Antiproliferative Effects of Isoalantolactone in Prostate Cancer Cells

**Figure 1:**
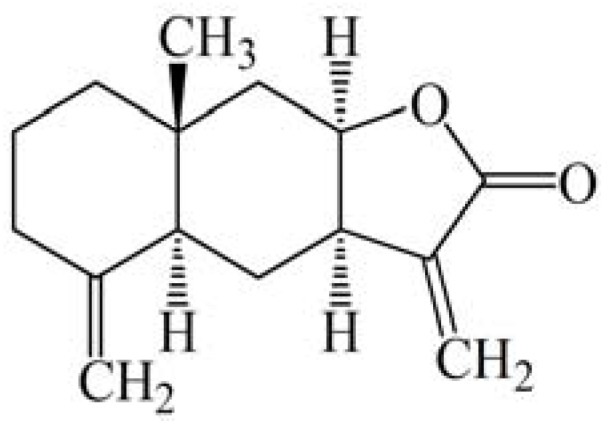
The chemical structure of isoalantolactone.

**Figure 2:**
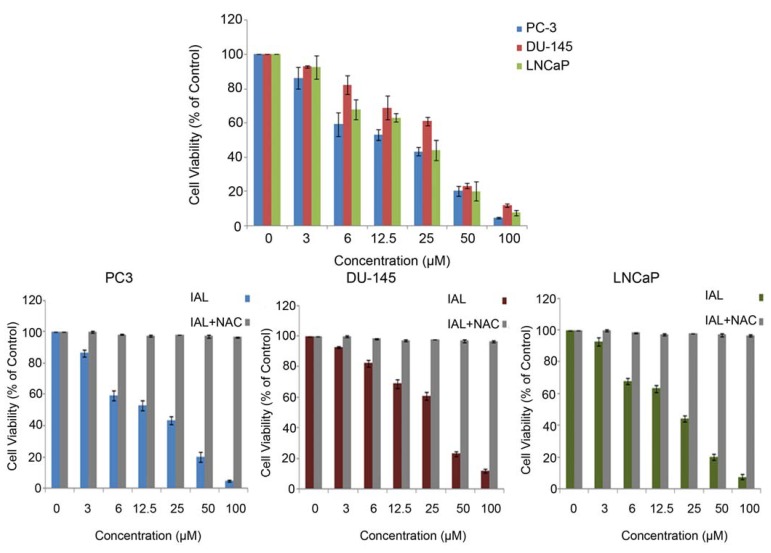
Isoalantolactone inhibited the cell growth and induced cell death in prostate cancer cells. LNCaP, PC-3 and DU-145 cells were treated with indicated doses of isoalantolactone in the presence or absence of NAC for 24 h and cell viability was measured by MTT assay. Data are expressed as Mean ± SD (n = 3).

Prevailing treatment options have had limited therapeutic success for androgen-independent prostate cancer cells. Hence, the current therapy for prostate cancer is not satisfactory and better therapeutic options are immediately required to develop a more effective therapy for this disease. Therefore, it is important to identify agents that induce death of both androgen-sensitive and androgen-independent prostate cancer cells. To identify a novel inducer of apoptotic cell death in prostate cancer cells, natural compounds were screened using the MTT assay. Isoalantolactone (Figure 1), isolated from the root of *Inula helenium*, was identified as a potent growth inhibitor of both androgen-responsive (LNCaP) as well as and androgen-insensitive (PC3 and DU-145) prostate cancer cells in a dose-dependent manner (Figure 2). As results were evaluated, the IC_50_ values in the PC3, DU-145 and LNCaP cell lines were 27.84 ± 3.34, 33.84 ± 4.06 and 29.84 ± 3.76, respectively. To further investigate the role of ROS in isoalantolactone-induced apoptosis in prostate cancer cells, we examined the effects of isoalantolactone in prostate cancer cells (LNCap, PC3 and DU-145), in the presence of NAC (5 mM), a specific ROS scavenger, using the MTT assay.

The treatment with isoalantolactone for 24 h inhibited the proliferation of prostate cancer cells in a dose-dependent manner. Pretreatment with the ROS scavengers NAC (5 mM) could almost completely eliminate the cytotoxicity of isoalantolactone and rescued the viability of cells indicating that isoalantolactone exerts cytotoxic effects on cell viability through ROS generation (Figure 2).

### 2.2. Isoalantolactone Induced Morphological Changes and Cell Death in Prostate Cancer Cells

**Figure 3:**
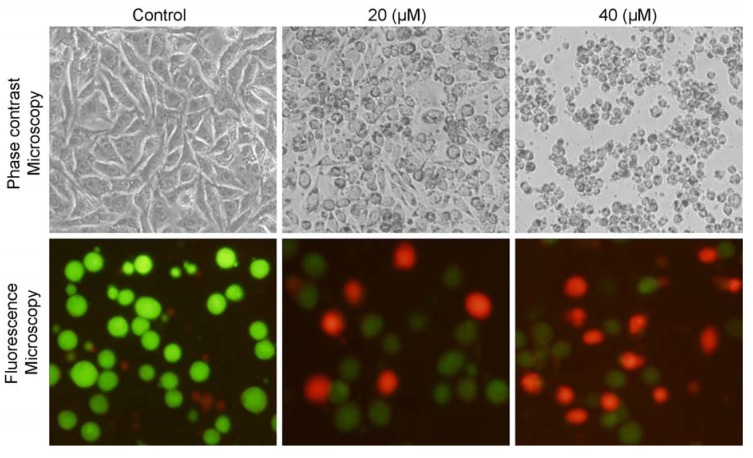
Morphological changes in human prostate cancer PC3 cells were observed under phase contrast and fluorescence microscopy after treatment with 0, 20 and 40 μM of isoalantolactone for 24.

Morphological changes were observed under microscopy after treating cells with isoalantolactone resulting in the decreased number of cells as compared to control group and cells became rounded and shrunken, whereas untreated cells were polygonal (Figure 3). Furthermore, the antiproliferative effect of isoalantolactone on PC3 cells was confirmed by a live/dead assay. For this purpose, prostate cancer PC3 cells were treated with 20 and 40 µM of isoalantolactone for 24 h and live and dead cells were observed using the fluorescent probe calcein AM/PI and fluorescence microscopy photographs were taken (Figure 3). The results demonstrated that treatment of cells with isoalantolactone decreased the viability of PC3 cells in a dose-dependent manner. Isoalantolactone thus induced growth inhibition of PC3 cells, in addition to other type of cancer cells previously reported including leukemia [12] and pancreatic carcinoma cells [13].

### 2.3. Isoalantolactone Induced G2/M Cell Cycle Arrest in PC3 Cells

**Figure 4:**
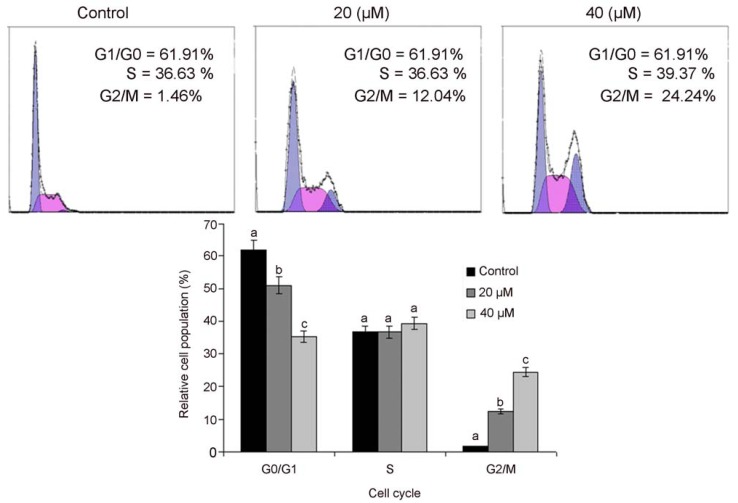
Flow cytometry analysis of cell cycle phase distribution in PC3 cells treated with 20 and 40 µM isoalantolactone for 24 h. Data are expressed as mean ±SD (n = 3). Columns not sharing the same superscript letter differ significantly (*p* < 0.05).

Recent insights related to cell cycle regulation indicated that cell cycle progression is tightly controlled by various checkpoints in normal cells while alterations the checkpoints of cell cycle progression lead to aberrant cell proliferation and development of cancer [14]. Tumor cells frequently acquire defects in the checkpoints resulting in the deregulation of cell cycle, which lead to unrestrained proliferation. Pharmacological correction of these check points and proper progression of cell cycle is a proficient strategy to control the growth and proliferation of cancer cells [15,16,17], so next, we analyzed the effects of isoalantolactone on cell cycle progression of PC3 cells. It was observed that isoalantolactone arrested cell cycle at the G2/M phase and the percentage of accumulation of cells in the G2/M phase was increased from 1.46 ± 0.46% in the control group to 12.64 ± 2.34% and 24.32 ± 1.98% in the cells treated with 20 and 40 µM of isoalantolactone for 24 h, respectively. This increase was coupled with a decreased percentage of cells in G0/G1 phase (Figure 4). These findings are also in line with reported results in pancreatic cancer cells in which isoalantolactone induced G2/M-phase cell cycle arrest [13]. The results clearly reveal that isoalantolactone induces cell cycle arrest in PC3 cells in the G2/M phase, therefore, isoalantolactone-induced cell death may result from cell cycle arrest and our investigation demonstrates that the isoalantolactone-induced cell cycle arrest of PC-3 cells appears to be one of the mechanisms of isoalantolactone’s anticancer activities. Recent studies documented that numerous chemotherapeutic and chemopreventive agents show potential anti-proliferative effects by arresting cell division at certain checkpoints in the cell cycle [18,19,20].

### 2.4. Effect of Isoalantolactone on Apoptosis in Human Prostate Cancer Cells

Apoptosis, autophagy, and necrosis are the major types of cell death [21]. Among these three major cell death pathways, apoptosis is the most well planned and orderly mode of cell death [22,23]. More than 40% of neoplasms undergo aberrations in the apoptotic machinery which leads to abnormal cell proliferation [24]. The regulation of apoptosis is, therefore, the most important in the treatment of cancer [25,26,27]. Accumulated evidence indicates that most chemotherapeutic agents halt tumor cell proliferation via induction of apoptosis [18,19,20].

**Figure 5:**
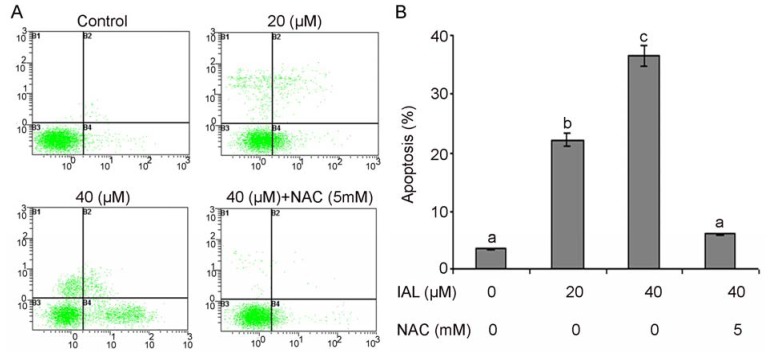
Apoptosis induced by isoalantolactone in PC3 cells. (**A**) PC3 cells were treated with 20 and 40 µM of isoalantolactone (IAL) for 24 h in the presence or absence of NAC. Then cells were stained with FITC-conjugated Annexin V and PI for flow cytometric analysis. The flow cytometry profile represents Annexin V-FITC staining in *x* axis and PI in *y* axis. (**B**) Data are expressed as Mean ± SD (n = 3). Columns not sharing the same superscript letter differ significantly (*p* < 0.05).

We examined whether isoalantolactone inhibited growth of PC3 cells through the induction of apoptosis. Isoalantolactone-induced apoptosis was determined by flow cytometric analysis. Cells were seeded in 12 well plates. After incubation of cells with 20 and 40 µM or without isoalantolactone for 24 h, cells were collected in centrifuge tubes and stained with annexin V-FITC and PI double staining as described in the Experimental section. Here, we found that that isoalantolactone is capable of inducing apoptosis in human prostate cancer PC3 cells. The flow cytometry analysis results showed that the rates of apoptosis were 22.13 ± 1.48% and 34.87 ± 1.34% in the cells treated with 20 and 40 µM of isoalantolactone, respectively, after 24 h, as compared to the 3.41 ± 0.52% in control cells (Figure 5A,B). Many studies have demonstrated that ROS can induce apoptotic cell death in various types of cancer cells after treatment with anticancer drugs [13,28]. To determine whether ROS plays an important role in isoalantolactone-induced apoptosis in PC3 cells, we pretreated cells with the ROS scavenger NAC at 5 mM for 2 h, and then we treated the cells with isoalantolactone for an additional 24 h. Pretreatment with NAC markedly abrogated the apoptotic effect of isoalantolactone indicating that induction of apoptosis is ROS-dependent (Figure 5A,B). Therefore, these results suggest that intracellular ROS plays an essential role in isoalantolactone-induced apoptotic cell death in PC3 cells, which was consistent with our observed cell survival data and compatible with previously reported studies [13].

### 2.5. Isoalantolactone Promoted ROS Generation in Prostate Cancer Cells

ROS are well known mediators of intracellular cascade signaling. The excessive generation of ROS can induce oxidative stress, loss of cell functionality, and apoptosis [29]. ROS can also be involved in the process of lipid peroxidation and or the cross-linking of thiol groups in proteins; both of these processes can induce the opening of the mitochondrial permeability transition pore (PTP) [30,31]. In the present study, we assumed that isoalantolactone might increase ROS levels, which could be involved in isoalantolactone-induced apoptosis. Therefore, the intracellular ROS level was measured using the ROS-detecting fluorescence dye 2,7-dichlorofluorescein diacetate (DCF-DA) because the DCF assay is highly sensitive, linear, and precise for measuring oxidative stress in irradiated cells [32]. The level of ROS was significantly increased in a dose-dependent manner after treating the cells with isoalantolactone, thus our results indicate that ROS generation were effective diminished by pretreatment with ROS scavengers when cells were treated with isoalantolactone.

**Figure 6:**
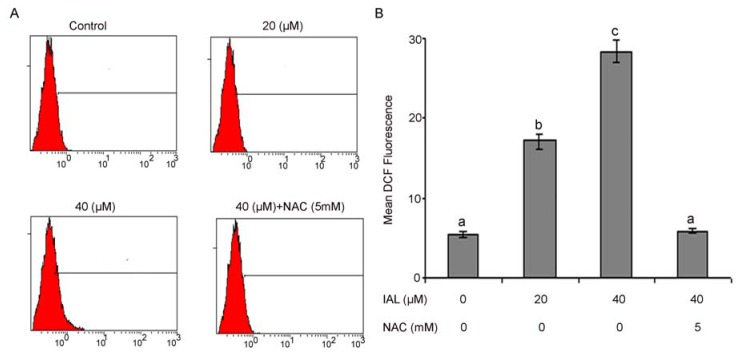
Flow cytometry analysis of ROS generation. (**A**) PC3 cells were treated with 20 and 40 µM isoalantolactone (IAL) in the presence or absence of 5 mM NAC for 24 h. (**B**) Data are expressed as Mean ± SD (n = 3). Columns not sharing the same superscript letter differ significantly (*p* < 0.05).

As shown in Figure 6A,B, the ratio of DCF-positive cells, treated with 20 and 40 µM isoalantolactone was significantly higher (18.37 ± 1.65 & 28.80 ± 1.83 *vs.* 6.54 ± 0.62 in control group, *p* < 0.05). These findings evidenced that isoalantolactone enhanced the generation of ROS in PC3 cells. The chemotherapeutic agents causing enhancement in oxidative stress are likely to be toxic to the cancer cells because they are found to be involved in biological processes like cell cycle arrest, DNA repair, and apoptosis [33].

### 2.6. Isoalantolactone Decreased Mitochondrial Membrane Potential in PC3 Cells

**Figure 7:**
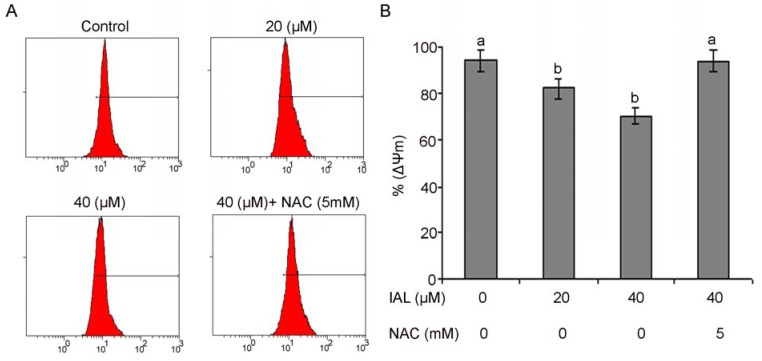
The effects of isoalantolactone on mitochondrial transmembrane potential of PC3 cells were determined by flow cytometry. (**A**) The values indicate the percentages of rhodamine 123 fluorescence in the PC3 cells treated without and with 20 and 40 µM of isoalantolactone (IAL) for 24 h in the presence or absence of NAC. (**B**) Data are expressed as Mean ± SD (n = 3). Columns not sharing the same superscript letter differ significantly (*p* < 0.05).

Mitochondria are an important component of the apoptosis execution machinery, which involves pro-apoptotic proteins (e.g., cytochrome c) [22]. It has been elucidated that depolarization of the mitochondrial membrane potential results in mitochondrial swelling and subsequent release of cytochrome c from the intermitochondrial membrane space into the cytosol [34]. It is becoming increasingly apparent that the mitochondria play a fundamental role in the processes leading to cell death [35]. The effects of isoalantolactone on the mitochondrial membrane potential of PC3 cells were determined by flow cytometry using rhodamine 123 staining. The rates of depletion of mitochondrial membrane potential were 82.12 ± 1.33% and 70.27 ± 1.74%, respectively, in the cells treated with 20 and 40 µM of isoalantolactone for 24 h as compared to 94.42 ± 0.53% in the control group. To further confirm the involvement of ROS in the disruption of mitochondrial membrane potential, cells were treated with 5 mM NAC. Pretreatment with NAC completely prevented dissipation of mitochondrial membrane potential, indicating that this was ROS-dependent (Figure 7A,B).

### 2.7. Isoalantolactone Regulated Apoptosis-Related Proteins in Prostate Cancer Cells

**Figure 8:**
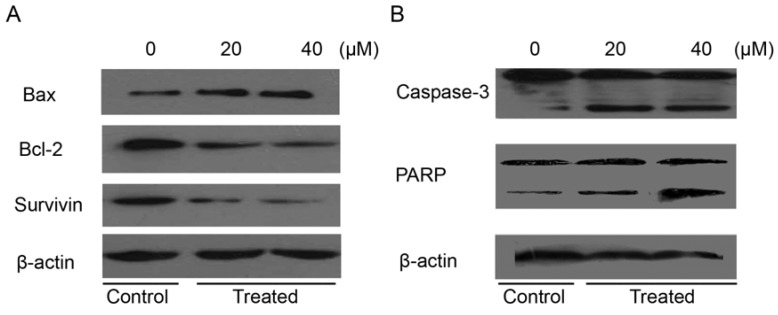
The effect of isoalantolactone on the expression of major apoptosis regulatory proteins. PC3 cells were exposed to 20 and 40 µM of isoalantolactone for specified time intervals. Equal amounts of lysate protein were subjected to gel electrophoresis. (**A**,**B**) Expression levels of Bax, Bcl-2, survivin, caspase-3 and PARP were monitored by western blot assay. β-actin was used as loading control. Data are representative of three independent experiments with similar results.

Our data corroborate the previously reported results that isoalantolactone induced dissipation of mitochondrial membrane potential, which provides evidence for a direct contribution of mitochondria in the isoalantolactone-induced apoptosis [13]. Interplay between pro-apoptotic (Bax) and anti-apoptotic (Bcl-2) members of the Bcl-2 family drives the mitochondrial apoptotic pathway [36]. Bcl-2 family proteins are pivotal for increasing the permeability of mitochondrial membranes and the release of cytochrome c, which activates caspases and in turn mobilizes apoptotic cell death [37,38,39]. To investigate the effect of isoalantolactone on expression of Bcl-2, western blotting was done. It was observed that isoalantolactone was involved in the down regulation of Bcl-2 in a dose-dependent manner (Figure 8A). These results are consistent with previously reported studies [12,13].

Furthermore, many studies have reported that hormone resistance of prostate cancer develops, in part, from up-regulation of antiapoptotic genes after androgen deprivation. Given the accumulating evidence that survivin, a new member of the inhibitor of apoptosis (IAP) family, is associated with both cancer progression and drug resistance, we hypothesized that survivin plays a potentially important role in hormone therapy resistance, and that targeting of survivin may enhance sensitivity to antiandrogen therapy in prostate cancer. Patterns of survivin expression were assessed in three prostate cancer cell lines LNCaP, PC-3, and DU-145 using quantitative western analysis. All three cell lines were found to strongly express survivin [40]. Therefore, these cumulative observations have validated survivin as a novel target for prostate cancer therapy, and hence we examined the effect of isoalantolactone on survivin, an anti-apoptotic protein. Our results reveal that isoalantolactone was involved in the down regulation of survivin in a dose-dependent manner in prostate cancer PC3 cells (Figure 8A).

The caspases are a family of proteins related to cysteine proteases that are one of the focal executors of the apoptotic process via triggering of the death receptors and mitochondrial pathways to accomplish programmed cell death [41]. Caspases are present in the form of inactive zymogens which are activated during apoptosis. Among them, caspase-3 is a frequently activated death protease, catalyzing the specific cleavage of many key cellular proteins [42]. In order to reveal the effect(s) of isoalantolactone on expression of caspase-3 and its downstream target, PARP, western blotting was done. The results showed that procaspase-3 was cleaved to yield 17 and 20 KDa fragments and activation of PARP in treated cells with 20 and 40 µM of isoalantolactone after 24 h as compared to that of control cells (Figure 8B). These findings are supported by previous studies [13]. These results confirm that isoalantolactone induced caspase-dependent cell death in PC3 cells.

## 3. Experimental

### 3.1. Chemicals and Reagents

Isoalantolactone was purchased from Tauto Biotech Co., Ltd. (Shanghai, China). Cell culture medium reagents and MTT [3′-(4,5-dimethylthiazol-2-yl)-2,5-diphenyltetrazolium bromide], propidium iodide (PI), and dimethyl sulfoxide (DMSO) were purchased from Sigma (St. Louis, MO, USA). Fetal bovine serum (FBS) was purchased from the Hangzhou Sijiqing Biological Engineering Materials Co., Ltd. (Hangzhou, China) An annexin V-FITC apoptosis detection kit was purchased from Beyotime Institute of Biotechnology (Shanghai, China). Rabbit polyclonal anti-human Bcl-2, Bax, survivin, PARP and cleaved caspase-3 antibodies were purchased from Wuhan Boster Biological Technology Co., Ltd. (Wuhan, China). Mouse anti-β-actin and anti-rabbit antibodies were purchased from Santa Cruz Biotechnology (Santa Cruz, CA, USA). Ponceou and cell lysis buffer for western blots and IP were purchased from Bio SS Beijing (Beijing, China). Rhodamine 123 was purchased from Invitrogen (Eugene, OR, USA).

### 3.2. Cell Culture

Human prostate cancer PC3, DU-145 and LNCaP cells were propagated in IMDM, DMEM, and DMEM and T-medium (1:1) nutrients mixture respectively supplemented with 10% FBS and antibiotics at 37 °C in a humidified atmosphere with 5% CO_2_ and 95% air. Cells were seeded in a 10 cm culture dish and allowed to grow to approximately 70% confluence before experimentation.

### 3.3. Cell Proliferation Assay

The cytotoxic effects of the isoalantolactone on the cells were determined by the MTT assay. Briefly, LNCaP, DU-145 and PC3 cells were seeded at a density of 1 × 10^4^ cells per well in 96-well plates and allowed to grow overnight. Cells were incubated with 100 µL of complete culture medium containing 0, 3, 6, 12.5, 25, 50, and 100 µM of isoalantolactone. After incubation for 24 h, growth of cells was determined by adding 10 µL MTT (5 mg/mL in phosphate buffered saline) to each well and incubated for 4 h. After removal of the medium, 150 µL DMSO was added to each well and shaken gently and carefully. The absorbance was read at a wavelength of 490 nm in a plate reader (ELX 800, BIO-TEK Instruments Inc., Winooski, VT, USA).

### 3.4. Morphological Observation under Phase Contrast and Fluorescence Microscope

PC3 cells were seeded in 12-well flat bottom microtiter plates and then treated with isoalantolactone at the concentration of 0 20, and 40 µM, respectively. After 24 h of treatment, the morphology of PC3 cells was observed under a phase contrast microscope. Furthermore, cells were stained with calcein acetoxymethyl ester (calcein AM)/PI in the dark for 20 min at room temperature and were observed under a fluorescence microscope (Olympus, Tokyo, Japan).

### 3.5. Flow Cytometric Analysis of Cell Cycle

For cell cycle analysis, PC3 cells were seeded in 12-well plates and then treated with 20 and 40 µM of isoalantolactone for 24 h. After treatments, the percentages of cells in the different phases of cell cycle were evaluated by determining the DNA content after propidium iodide (PI) staining as we described previously [43,44].

### 3.6. Flow Cytometric Determination of Apoptosis

The rate of apoptosis of PC3 cells was examined by flow cytometry using annexin V-FITC/PI staining. Briefly, PC3 cells were cultured in 6-well plates and allowed to attach overnight. Cells were treated with 20 and 40 µM of isoalantolactone for 24 h. Then cells were collected, washed and resuspended in PBS. Apoptotic cell death was measured by double staining annexin V-FITC and PI using the annexin V-FITC apoptosis detection kit (Beyotime Biotechnology, Shanghai, China) according to the manufacturer’s instructions. Flow cytometric analysis was performed immediately after staining. Data acquisition and analysis were performed by flow cytometry using the Cell Quest software. 

### 3.7. Flow Cytometric Determination of Reactive Oxygen Species (ROS) in PC3 Cells

In order to determine the intracellular changes in ROS generation, PC3 cells were stained with 2',7'-dichlorofluorescein diacetate (DCFH-DA). The fluorescent dye DCFH-DA is cell membrane- permeable and is converted into the cell membrane impermeable nonfluorescent compound DCFH by intracellular esterases. Oxidation of DCFH by reactive oxygen species produces highly fluorescent DCF. The fluorescence intensity of DCF inside the cells is proportional to the amount of peroxide produced. Briefly, PC3 cells were treated with 20 and 40 µM isoalantolactone for 24 h. After treatment, cells were further incubated with 10 μM DCFH-DA at 37 °C for 30 min. Subsequently, cells were harvested, rinsed, re-suspended in PBS, filtered with 300 apertures and analyzed for 2',7'-dichlorofluorescein (DCF) fluorescence by flow cytometry (Beckman Coulter, Epics XL, Miami, FL, USA).

### 3.8. Flow Cytometric Determination of Mitochondrial Membrane Potential (ΔΨ_m_)

To probe the changes in ΔΨ_m_, PC3 cells were stained with rhodamine 123 (1 μM) after treatment of 20 and 40 µM of isoalantolactone for 24 h with control group. The fluorescence of rhodamine 123 was measured by flow cytometry with excitation and emission wavelengths of 488 and 530 nm.

### 3.9. Western Blotting

To reveal the mechanism of the apoptotic effect of isoalantolactone, western blotting was done for apoptotic-related proteins as previously described [33]. Briefly, PC-3 cells were incubated with 20 and 40 µM of isoalantolactone for the indicated time. Cells were trypsinized, collected in 1.5 mL centrifuge tubes and washed with PBS. The cell pellets were resuspended in lysis buffer and were lysed on ice for 30 min. After centrifugation for 15 min, the supernatant fluids were collected and the protein content of the supernatant was measured on a NanoDrop 1000 spectrophotometer (Thermo Scientific, Waltham, MA, USA). The protein lysates were separated by electrophoresis on 12% SDS-polyacrylamide gel and transferred to a PVDF membrane (Amersham Biosciences, Piscataway, NJ, USA). The membranes were soaked in blocking buffer (5% skimmed milk) for 2 h. To probe for BAX, Bcl-2, survivin, cleaved caspase-3, PARP and β-actin, membranes were incubated overnight at 4 °C with relevant antibodies, followed by appropriate HRP conjugated secondary antibodies and ECL detection.

### 3.10. Statistical Analysis of Data

For the statistical analysis of data, comparisons between results from different groups were analyzed with SPSS for Window Version 15.0. Student’s *t-*test was employed to determine the statistical significance of the difference between different experimental groups and control group. *p* < 0.05 value was defined as statistically significant. All experiments were repeated at least three times. Data were presented as mean ± standard deviation (S.D).

## 4. Conclusions

Although in our previous studies, we have shown that isoalantolactone inhibits the growth of human pancreatic cancer cells by inducing apoptosis, the current study is the first to describe the role of ROS in the induction of apoptosis in prostate cancer cells. In addition, in the present study, we observed that isoalantolactone markedly down-regulated the expression of survivin in PC3 cells, which, to our knowledge, is the first observation that isoalantolactone could down-regulate the expression of the anti-apoptotic protein survivin in PC3 cells and the pathway that we have described herein is novel and has not been elucidated before. To conclude, isoalantolactone induces cell death in PC3 prostate cancer cells via induction of ROS-mediated apoptosis. Analysis of apoptosis-related proteins in PC3 cells revealed that isoalantolactone induced the up-regulation of Bax and the parallel down-regulation of Bcl-2. This ultimately led to disruption of mitochondrial membrane potential (ΔΨ_m_) and the sequential activation of caspase-3 and its downstream substrate, PARP, leading to apoptosis. Based on our previous and present studies, we suggest that isoalantolactone may represent an emerging novel therapeutic agent for the treatment of human cancers. Further studies are required to support our observations of the anti-cancer potential of this compound.
